# The lifetime prevalence and impact of generalized anxiety disorders in an epidemiologic Italian National Survey carried out by clinicians by means of semi-structured interviews

**DOI:** 10.1186/s12888-021-03042-3

**Published:** 2021-01-20

**Authors:** Antonio Preti, Roberto Demontis, Giulia Cossu, Goce Kalcev, Federico Cabras, Maria Francesca Moro, Ferdinando Romano, Matteo Balestrieri, Filippo Caraci, Liliana Dell’Osso, Guido Di Sciascio, Filippo Drago, Maria Carolina Hardoy, Rita Roncone, Carlo Faravelli, Cesar Ivan Aviles Gonzalez, Matthias Angermayer, Mauro Giovanni Carta

**Affiliations:** 1 University of Cagliari, Cagliari, Italy; 2 Department of Neuroscience “Rita Levi Montalcini”, University of Turin, via Cherasco 15, 10126 Turin, Italy; 3 Department of Innovation Sciences and Technologies, University of Cagliari, Cagliari, Italy; 4 Mailman School of Public Health, Columbia University, New York, USA; 5 University of Roma La Sapienza, Rome, Italy; 6 University of Udine, Udine, Italy; 7 Department of Drug Sciences, University of Catania, Catania, Italy; 8 Oasi Research Institute-IRCCS, Troina, Italy; 9 University of Pisa, Pisa, Italy; 10 University of Bari, Bari, Italy; 11 Azienda Ospedaliera Brotzu, Cagliari, Italy; 12 University of L’Aquila, L’Aquila, Italy; 13 University of Florence, Florence, Italy; 14 Universidad Popular del Cesar, Valledupar, Cesar Colombia; 15 Center for Public Mental Health, Gosim, Austria

**Keywords:** Generalized anxiety disorder, Comorbidity, Quality of life, Community survey, Epidemiology

## Abstract

**Background:**

Generalized anxiety disorder (GAD) is one of the most reported diagnoses in psychiatry, but there is some discrepancy between the cases identified in community studies and those identified in tertiary care. This study set out to evaluate whether the use of clinicians as interviewers may provide estimates in a community survey close to those observed in primary or specialized care.

**Methods:**

This is a community survey on a randomly selected sample of 2338 adult subjects. The Advanced Neuropsychiatric Tools and Assessment Schedule (ANTAS) was administered by clinicians, providing lifetime diagnosis based on the DSM-IV-TR. Health-related quality of life (HR-QoL) was measured with the Short-Form Health Survey (SF-12).

**Results:**

Overall, 55 (2.3%) subjects met the criteria for GAD, with greater prevalence in women (3.6%) than in men (0.9%): OR = 4.02; 95%CI: 1.96–8.26. Up to 40% of those with GAD had at least another diagnosis of mood, anxiety, or eating disorders. The mean score of SF-12 in people with GAD was 32.33 ± 6.8, with a higher attributable burden than in other conditions except for major depressive disorder.

**Conclusions:**

We found a relatively lower lifetime prevalence of GAD than in community surveys based on lay interviewers and a structured interview. The identified cases of GAD showed a strong impact on the quality of life regardless of co-morbidity and high risk in women, suggesting a profile similar to the one identified from studies in primary and specialized care.

## Background

Generalized anxiety disorder (GAD) is one of the most reported mental disorders in primary care and emergency services [1]. Prevalence estimates varied widely across countries, with higher lifetime prevalence in high-income countries than in middle−/low-income countries (5% versus 1.5 to 3%) [2]. The fifth edition of the *Diagnostic and Statistical Manual* of Mental Disorders (DSM-5), describes the GAD as characterized by persistent, excessive, and unrealistic worry about everyday things, usually involving more than an area, such as finance, family, health, and the future [3]. Anxiety in GAD is difficult to control and is accompanied by many non-specific psychological and physical symptoms, like, among others, muscle tension, fatigue, sleep disturbances, difficulty in concentrating, and irritability (DSM-5 code: 300.02) [3]. This profile of symptoms corresponds to the profile described in the fourth edition of the DSM (DSM-IV) [4] and its text revision (DSM-IV-TR) [5], thus favoring the comparison of studies across time.

GAD manifests high comorbidity with mood and other anxiety disorders, up to 50% and over, depending on the disorder and sample [2, 6]. GAD is often comorbid with bipolar disorder (BD), and might be associated with a more severe BD course and increased risk of suicide [7]. Role impairment is common in people with GAD and may be severe [2], as well as the association with chronic non-psychiatric diseases [6].

The etiology of GAD is unknown. A combination of genetics, environmental factors such as adverse childhood experiences, somatic disorders (including diabetes), alcohol and substance use, and the impact of stressful life events is thought to contribute to the onset, the course, and the persistence of GAD across lifetime. Some, low quality, brain imaging studies support a role in the expression of GAD symptoms of areas related to decision making, memory, cognitive flexibility, emotion appraisal and regulation, and detection of threat [8, 9]. GAD imports a high cost-of-illness, in terms of health expenditure and lost productivity, which has been estimated to be increased by a factor of 2.60 (95%CI: 2.01–3.36) [10]. Only about half of those with GAD seek treatment [2]. Patients with GAD may benefit from pharmacotherapy [11, 12]. In clinical practice, a combination of benzodiazepines and antidepressants is often prescribed [11, 12]. However, current guidelines emphasize that benzodiazepines should be avoided for long-term management of GAD and should be restricted to short-term use for the risk of tolerance and dependence [13, 14]. Pregabalin and quetiapine can be prescribed for long-term treatment of GAD [15]. Besides pharmacotherapy, cognitive behavioral therapy has been proved to be effective for GAD [16], while physical activity [17] and the application of transcranial magnetic stimulation [18] or transcranial direct current stimulation [19] may help for decreasing symptoms in GAD.

Despite GAD being one of the most reported diagnoses in psychiatry, and the validity of the phenotype received some support [20], the autonomy of the diagnosis was questioned by the findings of some epidemiological surveys [21]. For example, some of the symptoms required for major depressive disorder (e.g.., sleep difficulties, fatigue, and decreased concentration) overlap with GAD ones (being easily fatigued, difficulty concentrating, sleep disturbance). Indeed, the symptoms of GAD overlap in a large proportion with those of many other psychiatric conditions and a very small percentage of people diagnosed with GAD do not show another mental health diagnosis (about 17%) [21]. This is against the expectation of zones of rarity between syndromes [22]. Autonomous entities should show identifiable discontinuities with related conditions, with mixed conditions expected to be rarer than the pure forms [23]. Eventually, the actual diagnostic algorithm of the GAD goes into a detailed list of exclusion criteria, from obvious ones (the exclusion of the physiological effects of a prescribed or abuse substance or of a medical condition) to a cumbersome list of other mental disorders that should be assessed and whose impact on the anxiety, worry, or physical symptoms should be excluded (e.g., among others, anxiety or worry about having panic attacks in panic disorder, negative evaluation in social anxiety disorder, reminders of traumatic events in posttraumatic stress disorder, physical complaints in somatic symptom disorder, having a serious illness in illness anxiety disorder). Such a kind of detailed evaluation can be done in epidemiological survey but it is less easily conducted in the clinical setting. Moreover, studies on clinical samples provide data somewhat inconsistent with epidemiological studies, e.g. in a special anxiety unit in Göttingen, Germany, the proportion of patients seeking help had about 50% a diagnosis of panic disorder (frequency in epidemiological surveys around 2–3%) and only 7.5% a diagnosis of GAD (around 4% in epidemiological surveys) [24, 25].

These inconsistencies might depend on the fact that the cases identified in community studies are not the same as those identified in tertiary care. Indeed, in a diagnosis in which a central symptom such as worries has a fundamental clinical relevance, the use of “lay” interviewers and structured interviews can flatten the clinical relevance of the symptom’s centrality in epidemiological surveys [26]. Conversely, in the clinical setting greater attention is paid to patients’ reporting of theirs worries. A competing explanation could be that clinicians that work in specialized and tertiary care centers may overlook milder, but still burdensome symptoms: they may actually underdiagnose more soft cases because their clinical judgment is biased towards more severe and complex mental problems. Several studies conducted in the primary medicine setting described cases of GAD, rare in general but more frequent in the elderly (unlike some epidemiological studies that found greater frequency among young people), and with a severe impairment of health-related quality of life (HR-QoL) regardless of comorbidity with other anxiety and depressive disorders [27, 28].

The purpose of this work is to estimate the prevalence of GAD in a nationwide Italian sample. The impact of GAD and its comorbidity in terms of HR-QoL will be quantified, too. In this study, clinicians such as interviewers and semi-structured interviews (instead of lay interviewers and structured interviews like most epidemiological studies) will be used, and this might lead to the identification of a GAD profile different from that of other epidemiological studies previously conducted [29–36].

## Methods

This is an observational cross-sectional study (community survey).

### Design and procedure

The study sample was selected by randomization after stratification in 8 cells (gender and age 18–24; 25–44; 45–64; > 64) from records of municipalities of six Italian regions (one urban, one suburban, and at least one rural municipalities each region). The selected regions were representative of geographic and socio-economic characteristics of the whole 20 Italian regions.

Trained physicians or clinical psychologists conducted the interview face to face at homes of the enrolled people. This study is a secondary research of a project whose main objective was to study the appropriateness of psychiatric diagnosis and use of prescribed drugs in the Italian population. Details on the sampling procedure and the characteristics of the sample can be found in the parent article [37].

### Study tools

The psychiatric interviews were conducted by means of a semi-structured tool, the Advanced Tools and Neuropsychiatric Assessment Schedule (ANTAS) [37]. The ANTAS is a computerized tool inspired to the Structured Clinical Interview for DSM-IV (SCID) [38]. The ANTAS produces mood, anxiety and eating disorders diagnosis according to the DSM-IV-TR [5] with high cross-validity and reliability with SCID [37]. All diagnoses of psychiatric disorders were estimated as lifetime prevalence according to DSM-IV-TR criteria.

The Mood Disorder Questionnaire (MDQ) [39, 40] was adopted to assess lifetime subthreshold hypomanic episodes. Despite low accuracy in screening DSM-defined cases of bipolar disorder [41], the tool is good at identifying subthreshold cases [42].

The 12 items Survey Short Form (SF–12) [43] was used to measure the HR-QoL. The HR-QoL is a construct encompassing the self-perception of physical and psychological health. It is currently utilized as whole outcome and of impairment indicator in chronic diseases [44].

### Statistical analysis

The odds ratio (OR) in univariate analysis for DSM-IV TR GAD diagnosis and age, gender and comorbidity with DSM-IV-TR diagnosed disorders, was calculated using a single group as pivot by each table. The statistical significance of the associations was measured with the χ^2^, with or without Yates correction. The SF-12 mean scores between groups were compared with Analysis of Variance (ANOVA) one-way statistic.

The attributable burden on impairing HR-QoL of GAD was measured as difference between mean score on the SF-12 in a sample drawn from the same community survey database of people without GAD and the mean score of SF-12 of people with GAD. For this measure, the “healthy” control sample was obtained matching and randomization by blocks. For each person with GAD, a cell was created including all the people without GAD in the database of the same age and gender, thus four people for each cell were selected. The burden in impairing of HR-QoL attributable to GAD was also compared to a similar measure obtained to other diseases in previous case-control studies, which were carried out with the same methodology [45–51].

#### Ethics

The study was approved by the by the ethical committee of the Italian National Health Institute (Rome) and conducted according to the Declaration of Helsinki and its revisions [52]. All participants signed a written informed consent. They all received an appropriate referral to primary (general practitioner) or tertiary care (local psychiatric services) in case they manifest symptoms related to the disorders under investigation.

## Results

Table 1 shows lifetime prevalence of GAD by sex and age, the overall lifetime prevalence in the sample was 2.3%, with a markedly higher frequency in women (3.6%) than in men (0.9%; OR = 4.02; 95%CI: 1.96–8.26) and a substantially stable frequency in age in both sexes.

**Table 1:** Lifetime Prevalence of GAD by sex and age

	N (%)	χ^2^	*p*	OR	95%CI
Men					
< 25	0 (0)	–			
25–44	3 (0.8)	0.331^a^	0.565	OR = Inf	NV
45–64	2 (0.6)	0.854^a^	0.356	OR = Inf	NV
> 64	4 (2.1)	0.288^a^	0.090	OR = Inf	NV
Overall men	9 (0.9)				
Women					
< 25	5 (3.6)				
25–44	17 (3.8)	0.007^a^	0.933	1.04	0.39–2.91
45–64	15 (3.1)	0.023^a^	0.879	0.79	0.28–2.24
> 64	9 (3.6)	0.001^a^	0.999	0.99	0.32–2.01
Overall women	46 (3.6)				
Women vs. Men		16.770	< 0.0001	4.02	1.96–8.26
Men and women	55 (2.3%)				

^a^with Yates’s correction

The lifetime prevalence found by our research (2.3%) is lower than the one found in all other studies that were conducted through structured interviews administered by lay interviewers (Table 2).

**Table 2:** Comparison of Lifetime Prevalence of GAD with past community surveys

Study	Lifetime GAD %	Tool / Diagnostic System
Italy (Present study)	2.3	ANTAS-SCID DSM-IV
ECA, Los Angeles [30]	4.1	DIS-DSM III
ECA, Durham, San Louis [30]	6.6	DIS-DSM III
NCS [31]	5.1	CIDI-DSM-IIIR
NCS-R [35]	5.7	CIDI-DSM-IV
Sesto Fiorentino (Italy) [33]	5.4 / 3.9	UM-CIDI DSM-IIIR/DSM-IV
Taiwan Urban [29]	3.7	DIS-DSM III
Taiwan Small Town [29]	10.5	DIS-DSM III
Taiwan Rural [29]	7.8	DIS-DSM III
Korea [34]	3.6	DIS-DSM III
ESEMED [32]	2.8	CIDI DSM-IV
Singapore [36]	1.6	CIDI DSM-IV

With just the exception of the study of Chang in Singapore (1.6%), and the European Study of the Epidemiology of Mental Disorders (ESEMeD) [32], with estimates of 2.8%, the other studies ranged from 3.6% in Korea [34] to 10.5% in a small town in Taiwan [29].

As far as comorbidity was concerned, people with at least another diagnosis of mood, anxiety or eating disorders were 22 out 55 (40%). The most frequent diagnoses in comorbidity were: major depressive disorders (20%, OR = 5.97; 2.99–11.95), panic disorder (16.4%; OR = 17.4; 7.56–38.40), and simple phobia (16.3%; OR = 9.93; 4.58–21.55) (Table 3).

**Table 3:** Comorbidity between GAD and other disorders

	Comorbid with GAD	χ^2^	*p*	OR	95% CI
Major Depressive Disorder	11 (20%)	32.69^a^	< 0.0001	5.97	2.99–11.95
Obsessive-Compulsive Disorder	4 (7.3%)	6.14^a^	0.013	4.14	1.52–12.80
Panic Disorder	9 (16.4%)	84.75	< 0.0001	17.04	7.56–38.40
Social Phobia	1 (1.8%)	0.53^a^	0.467	6.01	0.72–79.94
Simple Phobia	9 (16.3%)	50.41	< 0.0001	9.93	4.58–21.55
Post-Traumatic Stress Disorder	2 (3.6%)	3.26	0.071	3.55	0.82–15.40
Eating Disorders	2 (3.6%)	0.25	0.612	1.45	0.34–6.80
Binge Eating	2 (3.6%)	3.95^a^	0.046	6.11	1.35–27.56
Bipolar Spectrum (MDQ+)	3 (5.4%)	0.92	0.336	1.78	0.54–5.84

^a^With Yates’s Correction

*MDQ* Mood Disorder Questionnaire

The level of HR-QoL in people with GAD (measured as mean score of SF-12) was 32.33 ± 6.8, without differences in people with (*N* = 22; 30.4 ± 7.0) or without comorbidities (*N* = 33; 33.6 ± 6.7): F (1;53) = 2.90; *p* = 0.094. Overall, with the only exception of major depressive disorder, GAD showed an attributable burden higher to that observed for the other investigated disorders from the same database (Table 4).

**Table 4:** Attributable Burden in worsening Quality of Life due to GAD and comparison with other disorders. Attributable Burden = Quality of Life in a matching control group without GAD (matching controls 1/4 from the community) – Quality of Life of People with GAD

	SF-12 (Mean ± sd)	Attributable Burden due to Disorder	Comparison with GAD	Comparison GAD without comorbidity
Major Depressive Disorder [45]	33.8 ± 9.2	5.6 ± 3.6 (*N* = 37)	F (1;90) = 2.42 *P* = 0.123	F (1;68) = 1.13 *P* = 0.291
Eating Disorders [46]	34 ± 6.2	4.4 ± 6.6 (*N* = 60)	F (1;113) = 4.77 *P* = 0.032	F (1;91) = 0.17 *P* = 0.681
Panic Disorder [47]	35.5 ± 4.6	2.9 ± 0.9 (*N* = 123)	F (1;176) = 166.01 *P* < 0.0001	F (1;154) = 43.56 *P* < 0.0001
Simple Phobia [48]	35.8 ± 6.1	2.5 ± 2.4 (*N* = 54)	F (1;107) = 64.98 *P* < 0.0001	F (1;84) = 18.12 *P* < 0.0001
Post-Traumatic Stress Disorder [49]	36.3 ± 6.1	3.9 ± 1.0 (*N* = 26)	F (1;79) = 21.03 *P* < 0.0001	F (1;57) = 2.82 *P* = 0.099
Obsessive-Compulsive Disorder [50]	35.4 ± 6.9	2.9 ± 6.0 (*N* = 88)	F (1;141) = 17.39 *P* < 0.0001	F (1;119) = 3.36 *P* = 0.069
Agoraphobia [51]	35.2 ± 7.8	3.4 ± 3.6 (*N* = 35)	F (1;88) = 20.93 *P* < 0.0001	F (1;66) = 3.55 *P* = 0.064
GAD	32.3 ± 6.8	6.5 ± 2.8 (*N* = 55)		
GAD without comorbidity	33.6 ± 6.7	4.9 ± 2.9 (*N* = 33)		

However, if we consider the cases of GAD without comorbidity, the “attributable burden” in impairing HR-QoL becomes comparable between GAD to that of most of the other disorders considered, except for panic disorder and simple phobia that resulted less impairing.

## Discussion

This survey, conducted by clinical interviewers who employed a semi-structured interview, showed a lower frequency of GAD in a sample of Italian general population compared to all community surveys conducted recently with the use of lay interviewing and clinical interviews structured [29–35], with the only exception of the study of Chang in Singapore [36] and the ESEMeD study [32]. The Chang ‘study also showed an increase from 0.9 to 1.6% compared to a study conducted in Singapore a few years earlier [36].

It is worth noting that our study highlights lower rates than research conducted on samples that are culturally closer, such as those examined by Faravelli’s study in a center of Tuscany in Italy [33]. Compared to this study, people with GAD in our sample have a lower frequency of comorbidity with other mood, anxiety or eating disorders (40% vs 70% of the study by Faravelli et al. [33]), and are more frequently women (4/1 ratio instead of 2/1). Another peculiar characteristic of our sample is that the frequency is stable over time and there is no higher frequency in the youth population as otherwise highlighted in the other community surveys conducted with structured interviews [25].

The stability of rates in different age groups, resulting in a higher rate in the elderly population (comparing with other community surveys) and the increased frequency in women makes our sample closer to the profile of GAD described in the specialist medical setting and/or primary care [27, 28]. It must be noted that a disorder like GAD, which should have a long course, should accumulate its frequency over time and, therefore, it would be logical to expect the lifetime rates in the elderly to be high. But this certainly applies to a disabling and high-impact disorder, less to a mild disorder that tends to be forgotten more frequently, generating higher recalling bias rates [53]. Nevertheless, several investigations noted a high prevalence of GAD in elderly people, with estimates around 10% or above [54–56]. A fraction of these cases were late-onset cases of GAD triggered by recent adverse life events and by chronic physical or mental (depression) health disorders. Adversities during childhood and a history of mental problems in parents were also related to recent onset GAD [55].

The GAD profile highlighted in our sample confirms that it has a severe impact on the lives of individuals, even independently of co-morbidity with other disorders, which, consistent with the cases highlighted in the primary medicine setting, defines a very well-defined pathology. Our study, therefore, seems to confirm that there may be a more clinically relevant (and less extensive) nucleus of people suffering from GAD and that the research conducted with hyper-structured methodology and using lay interviewers may produce an improper enlargement of the number of disorders that it may include people who are not properly suffering from a clinically important condition. This can be confirmed not only by the mismatch between the profile in community surveys and in health care agencies (which can be determined by barriers to access care for milder cases, although this is unlikely about primary care) but above all from the paradox of a progressive decrease over time of the lifetime frequency by age group.

The use of trained clinician interviewers is the strength of this study, together with the application of a standardized tool in community-based samples that were representative of the socio-cultural characteristics of the entire national territory. Nevertheless, some limitations must be acknowledged. The target of the original study was the lifetime prevalence of people diagnosed within the bipolar spectrum, which was estimated to involve 4% of participants. However, GAD and other anxiety disorders have lower lifetime prevalence, thus we were somehow underpowered to estimates some comorbid associations. We also lack information on somatic comorbidities, which may be relevant in GAD and reinforce the symptoms of anxiety in the disorder [6, 57].

## Conclusions

Our community survey conducted with a methodology that used clinical interviewers and a semi-structured interview showed a relatively low GAD frequency in the community than in other community surveys based on lay interviewers and a structured interview. The characteristics of the GADs of our sample (as a strong impact on the quality of life regardless of co-morbidity and high risk in women) indicate a disorder with characteristics very similar to those identified from studies in primary care and specialized care agencies.

It should be noted that there is no undisputable gold standard about GAD and, given the essential differences between the focus and scope of the clinician-based and lay-administered assessment methods, it cannot be decided whether the prevalence estimates of this study are more precise than those that can be derived from epidemiological studies based on lay-administered assessment methods. Only a direct comparison of the methods may consent an answer to that.

## Data Availability

The dataset for this article is not publicly available because the agreement shared with the partners in the planning of the study, in the presentation for the assignment of the original grant and the request for authorization to the ethics committee was that the database (with anonymized records) would be available only under the review of the project leader as guarantor. Requests to access the dataset should be directed to Professor Mauro Giovanni Carta.

## Abbreviations

ANOVA: Analysis Of Variance
ANTAS: Advanced Tools and Neuropsychiatric Assessment Schedule
BD: Bipolar disorder
CI: Confidence interval
DSM-5: D*iagnostic and Statistical Manual* of Mental Disorders, fifth edition
DSM-IV-TR: Diagnostic and Statistical Manual of Mental Disorders, fourth edition, text revision
ESEMeD: European Study of the Epidemiology of Mental Disorders
GAD: Generalized anxiety disorder
HR-QoL: Health related quality of life
MDQ: Mood Disorder Questionnaire
NICE: National Institute for Health and Care Excellence
OR: Odds ratio
SCID: Structured Clinical Interview for DSM-IV
SF–12: 12 items Survey Short Form
